# Geographic Disparities in the Opioid Overdose Crisis: Access, Treatment, and Mortality

**DOI:** 10.7759/cureus.102184

**Published:** 2026-01-24

**Authors:** Ahmed A Al-Awadhi, Sai M Vegiraju

**Affiliations:** 1 Anaesthesiology, German-Yemeni Hospital, Sana'a, YEM; 2 General Surgery, Ysbyty Ystrad Fawr, Aneurin Bevan University Health Board, Ystrad Mynach, GBR

**Keywords:** buprenorphine, criminal justice, healthcare disparities, health disparities, health equity, health services accessibility, methadone, opioid use disorder, rural health, telemedicine

## Abstract

The opioid overdose crisis in the United States (U.S.) exhibits profound and persistent geographic inequalities, where place of residence is a key determinant of risk, access to care, and mortality. This narrative review synthesizes evidence on the spatial evolution of the epidemic-from its prescription opioid origins in Appalachia to the nationalized spread of illicit fentanyl and polysubstance use-revealing that mortality “hotspots” are direct manifestations of underlying social vulnerability, economic distress, and healthcare infrastructural deficits. We document a severe, multi-layered chasm in access to life-saving medications for opioid use disorder (MOUD), characterized by rural treatment deserts-geographic areas with a critical shortage of MOUD providers-for buprenorphine, structurally embedded barriers to methadone, and restrictive insurance policies.

The review further identifies the criminal justice system as a critical risk amplifier, where incarceration and the post-release period represent times of extreme overdose vulnerability due to systemic failures in care continuity. Finally, we examine telehealth as a promising yet precarious tool for expanding access, one that is itself constrained by digital divides and uncertain regulations. The evidence underscores that geographic disparities are not incidental but are produced by an interconnected system of policy, infrastructure, and inequity. Consequently, mitigating the crisis requires moving beyond one-size-fits-all approaches to implement coordinated, place-based strategies that dismantle treatment barriers, integrate harm reduction into justice systems, and ensure equitable access to evidence-based interventions across all communities.

## Introduction and background

Although recent provisional CDC data indicate a substantial national decline, with opioid-involved deaths falling approximately 34% from 83,140 in 2023 to 54,743 in 2024, the death rate remains critically high as synthetic opioids continue to dominate the illicit drug supply [1]. This promising trend, which represents the lowest annual overdose estimate since 2019, is geographically uneven and must be sustained; many historically high-burden regions and communities continue to face profound vulnerabilities [1].

This crisis is profoundly uneven. Consider, for example, a resident of a rural treatment desert who must travel hours for medication, or a person leaving incarceration whose risk of fatal overdose spikes catastrophically in the first weeks of freedom. These are not hypotheticals but documented realities of the crisis, where mortality clusters along geographic lines and a person’s ZIP code is a powerful determinant of risk. Historically, urban counties reported higher mortality, yet rural communities have experienced far steeper increases-up to 1,600% in the Midwest and 1,141% in the Northeast-owing to limited healthcare access, economic stress, and social isolation [2]. Substance patterns also vary geographically, with rural overdoses more frequently involving prescription opioids, while urban areas see greater involvement of heroin and synthetics [2].

National policy analyses emphasize that the opioid epidemic reflects a complex, interconnected system. Policy responses must therefore account for prescription supply, illicit markets, demand reduction, and harm reduction simultaneously [3]. Interventions that target only one segment-such as prescription supply-risk unintended consequences, including transitions to cheaper illicit opioids, underscoring the need for coordinated, multi-level strategies [3]. Understanding these geographic and systemic disparities is essential for designing targeted prevention approaches and improving equitable access to treatment across diverse communities.

**Figure 1:**
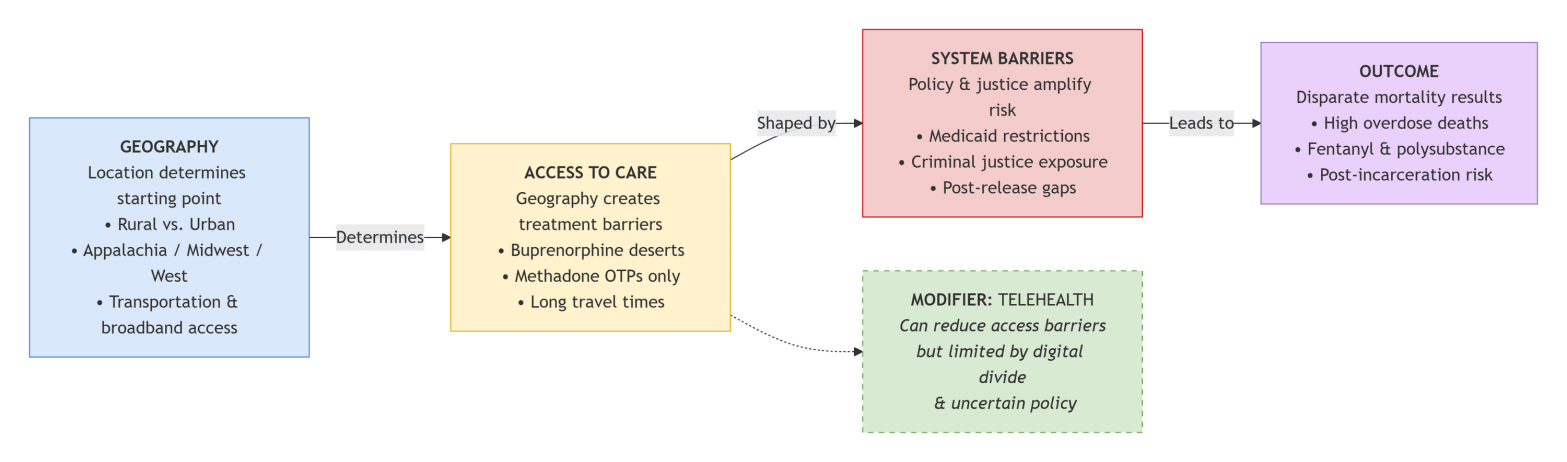
Conceptual Pathways Linking Geography to Opioid Overdose Mortality Disparities. Figure created by the first author (Ahmed A. Al-Awadhi). OTP: opioid treatment program

This narrative review synthesizes evidence to argue that geographic disparities in opioid overdose mortality are not incidental but are produced through sequential, systemic pathways. As illustrated in Figure 1, a conceptual model mapping the pathway from geographic context to mortality disparities, we propose a framework where a community’s geographic and social context determines access to life-saving interventions; this access is then filtered through restrictive policies and amplified by the criminal justice system, resulting in predictable spatial patterns of mortality.

This review will examine the evidence for each component of this framework, beginning with the evolving landscape of mortality, moving through the chasm of treatment access, and concluding with clinical and policy implications.

## Review

Methods

A thematic narrative review was conducted to synthesize evidence on geographic disparities in opioid overdose mortality, treatment access, and systemic barriers in the United States. A structured, iterative search strategy was employed to identify relevant literature across several thematic domains.

Targeted keyword searches were performed in PubMed, Science Direct, and Google Scholar, supplemented by hand-searching, without publication date restrictions. Search terms were combined to address core themes, including: (“opioid epidemic” OR “opioid crisis”) AND (“geographic disparity” OR “rural health” OR “urban health” OR “spatial analysis”); (“opioid use disorder” OR “OUD”) AND (“treatment access” OR “MOUD” OR “buprenorphine” OR “methadone”); and (“overdose mortality” OR “drug poisoning”) AND (“criminal justice” OR “incarceration” OR “telehealth” OR “health equity”). Hand-searching of reference lists from key articles and relevant reports from U.S. public health agencies (e.g., CDC and SAMHSA) supplemented the database searches. The final search and synthesis were conducted in December 2025 and updated in January 2025.

English-language U.S. studies-including original research, reviews, and policy analyses-that addressed spatial variation in opioid outcomes or access to care were considered. Commentary and non-U.S. studies were excluded. Identified literature was organized thematically to construct the conceptual framework presented in this review, which illustrates how geographic and structural factors systematically produce disparities in overdose mortality.

Principal findings

The Landscape of Mortality (the “Where People Die” Map)

The geographic distribution of opioid-related mortality has evolved through distinct, overlapping waves, creating a dynamic and complex national landscape. The early prescription opioid wave cemented Appalachia as a persistent epicenter, where economic dislocation and limited healthcare access created profound vulnerability, with mortality rates accelerating most rapidly in rural areas [4,5].

The illicit fentanyl wave, beginning around 2013, reconfigured this map. While it ultimately concentrated the highest absolute mortality rates in urban centers by 2020, its spread from the Northeast and Midwest to the Mississippi River region and the Western U.S. was rapid [6,7]. Notably, the crisis in Appalachia intensified across all community types, with rural counties sustaining the highest per capita death rates through 2021, fueled by poverty, constrained healthcare, and delayed harm reduction policy adoption [8].

This crisis has now merged with a fourth, polysubstance wave characterized by psychostimulants. County-level maps reveal a striking geographic convergence: high rates of psychostimulant-involved mortality that emerged in the Southwest and Midwest by 2018 had spread extensively through Appalachia and New England by 2020, closely mirroring established patterns of synthetic opioid mortality [8]. Research shows that counties with concurrently high opioid and psychostimulant mortality-clustered in Appalachia, the industrial Midwest, and the Mountain West-are strongly predicted by higher county-level social vulnerability [9].

This progression-from a regional prescription crisis to a nationalized fentanyl epidemic and finally a polysubstance crisis mapped onto community disadvantage-reveals that spatial risk is a manifestation of underlying social, economic, and policy inequities. Effective intervention thus requires place-specific strategies that address both the substances dominating the local drug supply and the root vulnerabilities that perpetuate the crisis.

The Chasm of Access: Treatment Deserts and Systemic Barriers

Substantial geographic disparities in access to medications for opioid use disorder (MOUD), particularly buprenorphine, are well-documented. Rosenblatt et al. [10] provided an early nationwide assessment, finding that in 2012, only 2.2% of U.S. physicians held a Drug Addiction Treatment Act (DATA) waiver and more than half of all counties (53.4%) had no waivered clinician, leaving over 30 million people-predominantly in rural areas-without local access. Waivered physicians were heavily concentrated in coastal metropolitan regions [10].

This geographic maldistribution is compounded by systemic underutilization. Andrilla et al. [11] found that 60.1% of rural counties lacked any active buprenorphine prescriber. Physicians who held waivers but did not prescribe cited barriers like limited time, low confidence, lack of specialty backup, and concerns about managing complex patients. Similarly, Jones and McCance-Katz [12] found underutilization was pervasive even outside rural settings, with low average prescriber caseloads and barriers such as perceived lack of patient demand and insurance challenges. Taken together, this evidence demonstrates that the United States faces both literal geographic treatment deserts and widespread systemic underutilization of available capacity, perpetuating limited access in rural and underserved regions.

Methadone Access and Geographic Inequality

While buprenorphine access is challenging, geographic barriers to methadone are more pronounced and structurally embedded. Federal law restricts methadone dispensing for opioid use disorder (OUD) to certified opioid treatment programs (OTPs), inherently limiting geographic availability.

National analyses highlight severe spatial disparities. Amiri et al. [13] found over 13.5 million people lived more than a 60-minute drive from the nearest OTP, compared to only ~846,000 for office-based buprenorphine. In rural areas, the mean travel time to an OTP was 56.5 minutes-over 40 minutes longer than to a buprenorphine provider. OTPs are concentrated in metropolitan cores, creating vast treatment deserts [13].

The clinical implications are significant. Rosenblum et al. [14] documented patients traveling 50-200 miles one-way or across state lines for methadone, a known risk factor for treatment dropout. This burden impacts outcomes even in urban areas; Alibrahim et al. [15] found a 10-20-minute commute reduced odds of methadone treatment completion by 33% compared to a sub-10-minute commute.

For those reliant on public transit, access is even more constrained. Kim et al. [16] introduced a “feels-like accessibility” metric incorporating walk, wait, and transfer times. They found 70% of a transit trip’s duration consisted of these components, and 26% of a proxy overdose victim population could not reach any OTP via transit within 180 minutes.

In summary, methadone access is constrained by a compounding set of barriers: a severe maldistribution of OTPs, prohibitive travel burdens that impair retention, and inadequate public transit access. These inequities underscore an urgent need for policy innovation to expand methadone dispensing beyond the traditional OTP model.

Medicaid and Policy Barriers

Geographic disparities in MOUD access are also rooted in structural inequities in insurance and policy. While Medicaid expansion increased coverage for low-income adults with substance use disorders, a national study found it did not translate into higher treatment utilization, indicating insurance alone is insufficient without improved system capacity [17].

This aligns with the National Academies’ consensus report, which states that despite MOUD’s efficacy, its accessibility remains severely limited by regulatory barriers (e.g., methadone confinement to OTPs and former buprenorphine waiver rules), sparse providers, and wide interstate variation in Medicaid coverage, including prior authorization and dose limits [18]. These intertwined regulatory, financial, and infrastructural barriers create treatment deserts that persist despite nominal insurance eligibility.

The Justice System Intersection: Incarceration as a Risk Vector

The systemic treatment barriers detailed above converge catastrophically with the criminal justice system. Incarceration acts as a powerful social determinant of health, and the transition from custody to community represents a peak moment of vulnerability to fatal overdose.

Individuals released from incarceration face a mortality risk substantially higher than the general population, driven overwhelmingly by overdose. A foundational study found the adjusted risk of death was 3.5 times higher for released inmates, spiking to 12.7 times higher in the first two weeks post-release, with the risk of fatal overdose 129 times higher in that period [19]. A scoping review confirms incarceration history is a significant risk factor, with the highest risk immediately after release [20]. The International Mortality After Release from Incarceration Consortium (MARIC), synthesizing data from over 1.3 million individuals, underscores the global scale of this public health issue [21].

Systemic Gaps and Enduring Disparities

These historical risks persist due to systemic treatment gaps within justice systems. A national analysis of over three million treatment admissions (2014-2021) found that while MOUD use in criminal justice-referred treatment has increased, a profound disparity remains. In 2021, only 33.6% of justice-referred individuals received MOUD, compared to 49.3% from other sources-a 15.6 percentage-point gap [22]. This disparity exists across most states, closing slowly.

Targeted programs demonstrate the potential for change. An evaluation of a jail-based MOUD program reported that 93.3% of participants initiating treatment in custody chose buprenorphine, and over half continued their prescription post-release [23]. It is critical to note that practices vary significantly across jurisdictions. While systemic gaps are widespread, such pioneering programs demonstrate that evidence-based care within correctional settings is both feasible and effective, providing a model for broader reform [23].

Conversely, the health consequences of carceral exposure are severe and community-wide. A nationally representative study found incarcerated individuals faced a 39% higher all-cause mortality risk and more than triple the overdose mortality risk. Furthermore, higher county incarceration rates were associated with increased mortality risks even for non-incarcerated residents, framing incarceration as a community-level public health determinant [24].

Beyond Bricks and Mortar: Digital and Telehealth Disparities

The effort to expand MOUD access now intersects with the digital transformation of healthcare. Telehealth, expanded during the COVID-19 pandemic, presents a low-barrier model for initiating buprenorphine, showing promise in engaging hard-to-reach populations, including those leaving incarceration [25]. Regulatory flexibilities allowing initiation via video or telephone have reduced transportation barriers, mitigated stigma, and facilitated same-day access [25,26].

However, telehealth introduces its own inequities. Adoption by U.S. substance use treatment facilities grew only modestly from 13.5% to 17.4% (2016-2019), with substantial state variation [27]. Critical digital divides persist, as rural residents, racial minorities, and low-income individuals face barriers like unreliable broadband, lack of smart devices, and low digital literacy [25]. These “feels-like” digital barriers can render services as inaccessible as physical travel [16]. Furthermore, telehealth does not solve downstream pharmacy deserts or ensure care continuity if individuals are transferred to non-MOUD correctional facilities [23].

The policy landscape remains precarious. Evidence synthesizes that telehealth for buprenorphine is feasible, supports retention, and is preferred for its flexibility [25,26]. Yet, the permanent regulatory future is uncertain. Reinstating the pre-pandemic in-person requirement would re-erect a major barrier to care [26]. The long-term impact of telehealth on retention and mortality outcomes, especially in rural and justice-involved populations, remains a critical area for ongoing evaluation [25,27]. Sustaining equitable telehealth access, therefore, requires making regulatory changes permanent while implementing parallel policies to bridge the digital divide. Without this comprehensive approach, telehealth risks creating a new frontier of disparity.

Clinical and policy implications

The evidence reviewed underscores that the opioid crisis is not a monolithic epidemic but a constellation of localized outbreaks shaped by intersecting geographic, systemic, and social vulnerabilities. This necessitates a fundamental shift from uniform, one-size-fits-all interventions to tailored, place-based strategies. Clinically, this means treatment protocols must account for the local drug supply (e.g., fentanyl vs. prescription opioids vs. polysubstance use) and the specific barriers faced by the community (e.g., transportation deserts, digital divides, and high incarceration rates). For policymakers, the implication is that reducing overdose mortality requires coordinated action across multiple domains.

Treatment Infrastructure

Permanently remove regulatory barriers to MOUD provision, such as sustaining telehealth flexibilities and expanding methadone dispensing beyond OTPs. Policies must incentivize and support clinicians in rural and underserved areas to provide MOUD.

Criminal Justice Integration

Evidence-based overdose prevention must be integrated into all points of the justice system. This includes providing MOUD and naloxone in jails and prisons, ensuring warm handoffs to community care upon release, and mandating MOUD as a standard of care in court-referred treatment.

Equitable Access

Interventions must actively confront the underlying inequities that create treatment deserts. This requires investing in broadband and digital literacy to make telehealth viable, expanding public transit access to OTPs, and enforcing parity in Medicaid coverage for MOUD across all states.

Future research directions

To translate the well-documented geographic and systemic disparities into effective action, future research must prioritize implementation science and precision public health. A primary focus should be on developing and testing scalable models for delivering evidence-based interventions-such as mobile treatment units, integrated care in rural clinics, and comprehensive jail-based MOUD programs-within diverse, high-risk community settings. Employing hybrid effectiveness-implementation designs will be crucial to simultaneously evaluate health outcomes and identify the contextual determinants of sustainable adoption.

Concurrently, a more nuanced understanding of intervention effectiveness across different populations and policy environments is needed. Comparative effectiveness research should investigate whether treatment modalities (e.g., telehealth vs. in-person and different MOUD medications) yield differential outcomes for key subgroups, including justice-involved individuals and those in polysubstance use transitions. Furthermore, the field requires rigorous, longitudinal policy evaluations to assess the real-world impact of structural reforms-such as the elimination of the X-waiver or Medicaid continuity initiatives-on treatment access, retention, and overdose mortality at the population level.

Finally, advancing predictive analytics and etiological insight is essential for proactive intervention. Research must leverage spatial modeling and integrated data systems to develop dynamic, community-level risk prediction tools that account for evolving drug supplies and social vulnerabilities. Complementing this, mixed-methods studies are needed to elucidate the underlying social and economic mechanisms driving the geographic synergy between opioid and stimulant epidemics, thereby informing more foundational and targeted prevention strategies. Furthermore, implementation science research should explicitly investigate how to overcome pervasive barriers-such as clinical workforce deficits, funding instability, and political resistance-that hinder the adoption of evidence-based, place-based interventions in diverse community settings.

## Conclusions

In conclusion, disparities in opioid overdose mortality are not accidental but are systematically produced by a landscape of uneven access to treatment, entrenched policy barriers, and the synergistic harms of the criminal justice system. The path forward requires dismantling these structural inequities through clinically informed, geographically intelligent, and justice-oriented policies. This necessitates targeted, place-based strategies that directly confront the persistent disparities in MOUD access and overdose burdens, which continue to disproportionately affect rural communities and underserved urban areas despite recent national declines in mortality. By aligning public health and clinical practice with the complex reality of place, it is possible to transform the crisis map from one of mortality to one of recovery and resilience.
